# Sensitivity to temporal structure facilitates perceptual analysis of complex auditory scenes

**DOI:** 10.1016/j.heares.2020.108111

**Published:** 2021-02

**Authors:** Lucie Aman, Samantha Picken, Lefkothea-Vasiliki Andreou, Maria Chait

**Affiliations:** a Ear Institute, University College London, 332 Gray's Inn Road, London WC1X 8EE, UK; b Department of Psychiatry, University of Cambridge, Cambridge, UK; c Vocational Lyceum of Zakynthos, Ministry of Education, Research and Religious Affairs, Zakynthos, Greece

**Keywords:** Temporal regularity, Auditory scene analysis, Change detection, Time perception, Change deafness, Predictive coding

## Abstract

The notion that sensitivity to the statistical structure of the environment is pivotal to perception has recently garnered considerable attention. Here we investigated this issue in the context of hearing. Building on previous work (Sohoglu and Chait, 2016a; elife), stimuli were artificial ‘soundscapes’ populated by multiple (up to 14) simultaneous streams (‘auditory objects’) comprised of tone-pip sequences, each with a distinct frequency and pattern of amplitude modulation. Sequences were either temporally regular or random.

We show that listeners’ ability to detect abrupt appearance or disappearance of a stream is facilitated when scene streams were characterized by a temporally regular fluctuation pattern. The regularity of the changing stream as well as that of the background (non-changing) streams contribute independently to this effect. Remarkably, listeners benefit from regularity even when they are not consciously aware of it. These findings establish that perception of complex acoustic scenes relies on the availability of detailed representations of the regularities automatically extracted from multiple concurrent streams.

## Highlights

• Perception relies on sensitivity to predictable structure in the environment.
• We used artificial acoustic scenes to investigate this in the auditory modality.
• Listeners track the temporal structure of multiple concurrent acoustic streams.
• Sensitivity to predictable structure supports auditory scene analysis, even when scenes are complex.
• Benefit of regularity observed even when listeners are unaware of the predictable structure.

Due to their idiosyncratic physical constraints, most animate objects produce statistically structured, temporally predictable, sensory signals (e.g., vocalizations, locomotion). Accumulating evidence across sensory modalities, suggests that observers are sensitive to this patterning and use it to understand, and efficiently interact with, their surroundings (e.g., Rohenkohl et al., 2012; Leaver et al., 2009; Andreou et al., 2011; Yaron et al., 2012; Nelken, 2012; Costa-Faidella et al., 2011; Winkler et al., 2009).

The ability to extract and track temporal regularities should perhaps be particularly relevant in the auditory modality due to the dynamic and transitory nature of acoustic information. Indeed, the potential role of sensitivity to regularity in the context of ‘automatic’ auditory scene analysis is attracting considerable attention (Yaron et al., 2012; Nelken, 2012; Costa-Faidella et al., 2011; Winkler et al., 2009; Andreou et al., 2015). These investigations are motivated by demonstrations that listeners are acutely tuned to patterns in sound sequences (Bendixen et al., 2009; Bendixen, 2014; Barascud et al., 2016) and quick to form predictions about future input such that expected events are detected and assessed more rapidly and accurately than unexpected events (Leaver et al., 2009; Bendixen, 2014; Jones et al., 2002; Heilbron and Chait, 2018; but see Prince and Sopp, 2019).

Sensitivity to regularity has also been suggested to facilitate listening in crowded environments. The logic is that once the spectro-temporal regularities characterizing each object have been extracted, these patterns can then be used to segregate the input sound mixture by ‘pulling out’ sounds that conform to each ‘regularity rule’ and assigning them to their respective objects (Winkler et al., 2009). Accordingly, several studies (Andreou et al., 2011; Rimmele et al., 2012; Southwell et al., 2017) demonstrated that the temporal structure of a distractor sound sequence affects listeners’ ability to attend to another concurrently presented task-relevant sequence.

Overall however, the degree to which listeners detect and use auditory regularities remains poorly characterized. Many previous investigations have focused on very simple stimuli, consisting of just one or (occasionally) two concurrent sequences, and tasks that required listeners to explicitly attend to one of the elements in the scene - far from the challenges we regularly face in crowded, natural environments.

**Fig. 1:**
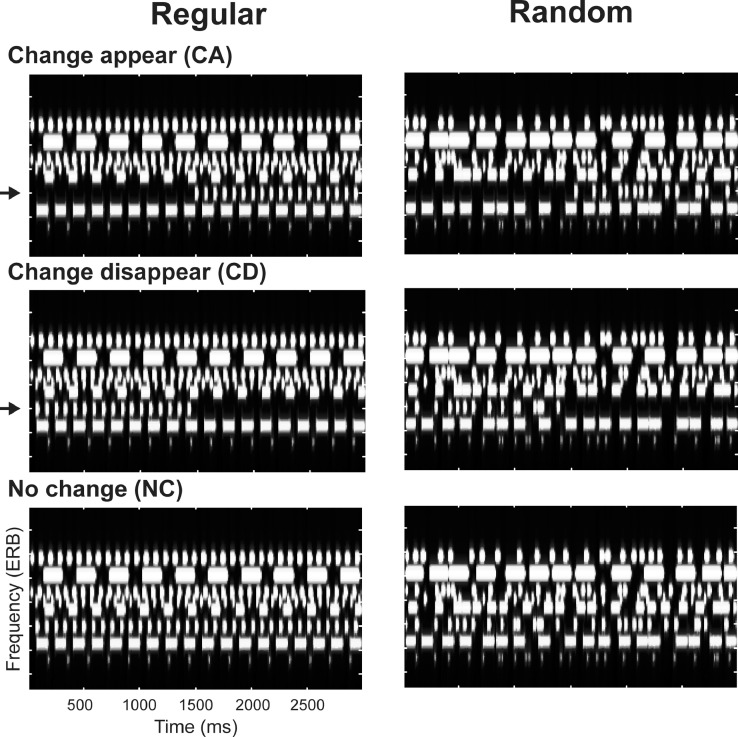
Example of the three variants (‘Change appear’, ‘Change disappear’, and ‘No change’) of a scene with 8 streams. Changing streams are indicated with arrows. Regular (REG) scenes are on the left, random (RAND) scenes on the right. The scenes are matched spectrally, with only temporal structure differing between REG and RAND scenes. The plots represent ‘auditory’ spectrograms, generated with a filter bank of 1/ERB wide channels equally spaced on a scale of ERB-rate. Channels are smoothed to obtain a temporal resolution similar to the Equivalent Rectangular Duration (Moore and Glasberg, 1983).

Sohoglu and Chait (2016a) demonstrated that sensitivity to regularity plays a role in complex listening conditions. A change detection task was used as an ecologically relevant means through which to probe sensitivity to predictable structure. Listeners were presented with artificial acoustic scenes (Fig. 1) comprising several concurrent sound-streams, each consisting of a sequence of tone pips of a particular frequency and with rates commensurate with those characterizing many natural sounds. Their task was to detect occasional changes (appearance of a stream) within those ‘soundscapes’. Keeping the spectral information matched, the temporal structure of the streams within the scene was manipulated such that they were either temporally regular (repeated with a fixed inter-pip-interval) or random (repeated with a random inter-pip-interval). Activity in auditory cortex rapidly (within 400 ms of scene onset) distinguished scenes comprised of temporally-regular (REG) vs. temporally-random (RAND) streams. Over and above this, the appearance of a stream in REG scenes evoked increased responses relative to RAND scenes. This mirrored the behavioural performance data which showed improved accuracy and quicker response times to appearance events in REG relative to RAND scenes. The results thus demonstrated that the auditory brain closely tracks the regularity of unfolding sound sequences and uses this information to facilitate responses to scene changes.

In the series of behavioural experiments reported below, we systematically manipulated acoustic scene properties, including the type of change and complexity of the regularity, in order to understand the factors that underlie listeners’ sensitivity to the temporal structure of complex soundscapes. Delineating this capacity is crucial towards understanding listening in complex environments and may also help explain failure of scene analysis in certain populations.

Sohoglu and Chait (2016a) investigated a specific instance of benefit from regularity where regular context was shown to facilitate the detection of an unexpected acoustic transient (see also Southwell and Chait, 2018). Here we extend this paradigm to other scene events, in particular stream disappearance, where the change event is not characterized by a frequency transient (Experiment 1). We also investigated whether the observed effects encompass more complex regularities than the simple isochronous patterns used previously (Experiment 2). Lastly, we aimed to determine whether the effect of regularity is driven by sensitivity to the temporal regularity of the changing (appearing or disappearing) **stream** per se, or by that of the **context** (the other, non-changing, streams in the scene; Experiment 3).

Following Sohoglu and Chait (2016a) we use a change detection paradigm as an objective means to assess the role of sensitivity to temporal regularity in auditory scene analysis. Unlike previous investigations which involved attention to a specific ‘foreground’ element, the stimuli here consist of many simultaneous streams, with listeners not explicitly monitoring a particular sequence but, rather, trying to make sense of the scene as a whole.

Overall the results demonstrate that listeners are exquisitely sensitive to temporal regularity and routinely extract and track the complex temporal structure of multiple concurrent acoustic streams. This can be observed even in very crowded scenes consisting of up to 14 independently temporally modulated sound sequences.

## 1 Experiment 1A – change detection is improved in REG relative to RAND scenes

Sohoglu and Chait (2016a) previously demonstrated that detecting item appearance in REG scenes is improved relative to RAND scenes. This was interpreted as indicating that listeners extract and track the temporal structure of individual scene streams, rendering unexpected events, such as those associated with the appearance of a new stream, more surprising, and hence more salient, in REG relative to RAND scenes.

Here we investigate the same process in the context of item disappearance. Item disappearance relies on different computational and neural substrates than item appearance (Cervantes Constantino et al., 2012; Sohoglu and Chait, 2016b). In particular, whilst appearance is revealed by a sustained increase in spectral power associated with the new stream, disappearance must be inferred from a decrease in activation. Previous work has suggested that disappearance is, at least in part, detected through monitoring “second order” offset responses, which reflect the coding of an expected, but missing, event (Andreou et al., 2015; Cervantes Constantino et al., 2012; Sohoglu and Chait, 2016b). Therefore, if listeners indeed monitor the temporal structure of all components in the scene, they should also be faster and more accurate at detecting item disappearance in REG, relative to RAND, scenes. This is because optimal disappearance detection directly depends on an accurate representation of upcoming tone pips - an ideal observer can detect the cessation of a stream at the moment an expected tone pip fails to arrive. Importantly, since the identity of the disappearing stream is a-priori unknown, improved performance in REG scenes would require the ability to simultaneously track the temporal structure of all (or at least a large number of) scene components.

### 1.1 Materials and methods

#### 1.1.1 Stimuli

The artificial ‘sound-scapes’ used here simulate challenges faced by listeners in natural acoustic scenes, in which many concurrent sound streams, each with a distinctive temporal pattern, are heard simultaneously (Eramudugolla et al., 2005; Snyder et al., 2012). Unlike natural sounds, however, the present stimuli are designed such that streams occupy distinct spectral ranges and hence do not energetically mask each other. This enables us to (1) create the optimal conditions for the streams to be perceived as independent auditory objects, and (2) measure the effect of growing scene size (number of concurrent streams present) independently of increased inter-stream masking. Stimuli (Fig. 1) were 2000–4000 ms long artificial ‘scenes’ populated by multiple (4, 8 or 14) streams of pure-tones designed to model sound streams. Each stream is characterized by a different carrier frequency (drawn from a pool of fixed values spaced at 2*ERB between 100 and 4846 Hz), and a unique amplitude modulation rate (AM; square wave; such that each stream consists of a sequence of tone pips). In a previous series of experiments (Cervantes Constantino et al., 2012), we demonstrated that these stimuli are perceived as a composite ‘sound-scape’ in which individual streams can be perceptually segregated and selectively attended to, and are therefore good models for natural acoustic scenes.

It is important to note that in the present experiments the concept of scene size is confounded with scene density - the more streams (‘sources’) in the scene the closer they are to each other. This is an inevitable consequence of using a fixed frequency range for the scenes, but, importantly, the same constraint also characterizes the notion of scene size in the environment (where the limit may be imposed by the hearing range in humans). We continue to refer to the manipulation as ‘scene size’ to be consistent with previous work (Cervantes Constantino et al., 2012; Sohoglu and Chait, 2016b; Eramudugolla et al., 2005; Pavani and Turatto, 2008). Importantly, the restriction that streams are at least 2 ERB apart controls the issue of density to some extent in that the large spectral separation between neighboring streams minimizes peripheral masking, enabling the investigation of the effects of increasing scene size without the confound of increasing inter-stream sensory masking.

In the ‘regular’ scenes (REG), the duration of a tone pip (values uniformly distributed between 20 and 160 ms; ramped on and off with a 5 ms raised cosine ramp) and the silent interval between pips (values uniformly distributed between 2 and 160 ms) are chosen independently (and separately for each stream) and then fixed for the duration of the scene so that the pattern is regular (see Fig. 1, left column). This pattern mimics the regularly modulated temporal properties of many natural sounds. In ‘Random’ (RAND) scenes, tone duration remains fixed throughout the scene, but the silent intervals between successive pips are varied randomly (values uniformly distributed between 2–160 ms) resulting in an irregular pattern (See Fig. 1, right column).

Scenes in which each stream is active throughout the stimulus are referred to as ‘no change’ stimuli (NC). Additionally, we synthesized scenes in which a stream became active (appeared) or inactive (disappeared) at some intermediate time during the scene. These are referred to as ‘change appear’ (CA) and ‘change disappear’ (CD) stimuli, respectively. The timing of change varied randomly (uniformly distributed between 1000 ms and 2000 ms post scene onset), but with the following constraints: The nominal time of change for CA streams coincided with the onset of the first tone while for CD streams the nominal time of change was set to the offset of the last tone augmented by the inter-tone interval, i.e., at the expected onset of the next tone, which is the earliest time at which the disappearance could be detected. For disappearing streams in RAND scenes, it is impossible to define change time in this way (because there is no regular temporal structure). For the purpose of measuring RT, the CD change time in RAND scenes was set to the offset of the last tone-pip augmented by the mean inter-pip-interval (80 ms). Because the distribution of inter-pip-intervals was identical in REG and RAND conditions, if temporal structure does not play a role in change detection, RT should be identical in both conditions.

The set of carrier frequencies and modulation patterns was chosen randomly for each scene, but to enable a controlled comparison between conditions, NC, CA and CD stimuli were generated as triplets sharing the same carrier frequencies and modulation patterns (but differing by the appearance or disappearance of a stream; see Fig. 1). They were then presented in random order during the experiment, blocked by change type (NC and CA or NC and CD) and scene type (RAND or REG). Each block contained equal numbers of no change (NC) or change (CA or CD) scenes such that the occurrence of change (and change time) were unpredictable. Overall 45 trials of each stimulus condition were presented.

Stimuli were synthesized with a sampling rate of 44,100 Hz and shaped with a 30 ms raised cosine onset and offset ramp. They were presented with an EDIROL UA-4FX sound card (Roland Corporation) over headphones (Sennheiser HD 555) at a comfortable listening level (~60–70 dB SPL), self-adjusted by each participant. Stimulus presentation was controlled using the *Cogent*software (http://www.vislab.ucl.ac.uk/cogent.php).

#### 1.1.2 Procedure

The experiment was conducted in an acoustically-shielded booth (IAC, Winchester, UK). Experimental sessions lasted about 2 h and consisted of a short practice session with feedback, followed by the main experiment without feedback, divided into runs of approximately 10 min each. Subjects were instructed to fixate at a cross presented on the computer screen, and perform a change detection task whereby they pressed a keyboard button as fast as possible when they detected a change in the presented stimulus. They were allowed a short rest between runs.

#### 1.1.3 Analysis

Dependent measures were d’ scores and response times (RT). For calculating d’, hits were defined as responses to the target trials (CA or CD), that occurred after the change in the scene. False positives (fp) were defined as responses to NC trials, or responses in CA/CD trials which occurred before the time of change. The latter were exceedingly rare, likely because participants knew that scene changes occurred about partway through the trial and therefore withheld early responses. In situations where hit rate = 1 or fp rate=0 (leading to an undefined d’) a correction (adding/subtracting 1/2R; where R is the number of trials) was applied. RT were measured between the nominal time of change and the subject's key press. Repeated measures ANOVA was used for the main analyses. Only interactions involving the parameters of interest (regularity) were explored with post-hoc tests. The α level was *a priori* set to 0.05.

#### 1.1.4 Participants

Ten paid participants took part in the experiment (5 female; mean age = 25 years). All reported normal hearing and no history of neurological or audiological disorder. Experimental procedures (here and in subsequent experiments) were carried out in accordance with the protocols approved by the research ethics committee of University College London, and written informed consent was obtained from each participant. The sample size (10 participants; here and in subsequent experiments) is based on previous experience in the lab with similar paradigms. As can be seen below the effects are stable, consistent across participants, and yield high effect sizes (partial η^2^ >0.7). Indeed, a similar effect size (η^2^ =0.717) was observed using a very similar paradigm in de Kerangal et al. (2020) for a sample size of 100 participants, confirming that the sample size is appropriate.

### 1.2 Results

**Fig. 2:**
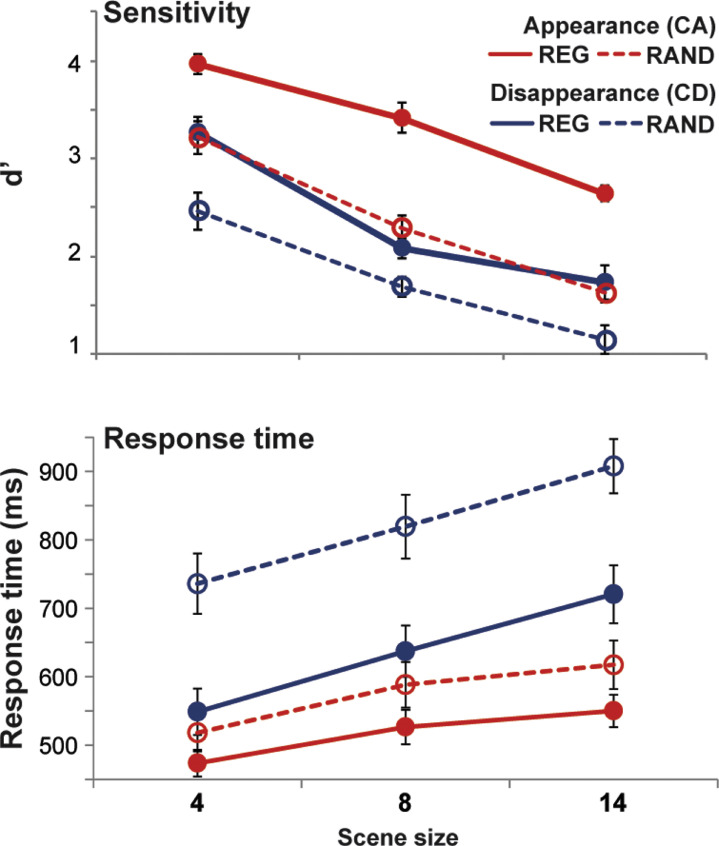
Results of Experiment 1. Error bars are 1 standard error (SE). In all measures (d’ and response time) performance is significantly reduced in RAND relative to REG scenes. (For interpretation of the references to color in this figure, the reader is referred to the web version of this article.)

Fig. 2 shows change detection performance for ‘appearing’ (CA; in red) and ‘disappearing’ (CD; in blue) events.

A repeated measures ANOVA on **d’ sensitivity scores** with scene regularity (REG vs. RAND), change type and scene size as factors revealed main effects of regularity (F(1,9)=86.52, *p*<.0001; partial η^2^ =0.906), change type (F(1,9)=47.25, *p*<.0001; partial η^2^ =0.840) and scene size (F(2,18)=244.01, *p*<.0001; partial η^2^ =0.964). We also observed an interaction between scene regularity and change type (F(1,9)=13.8, *p*=.005; partial η^2^ =0.605). An inspection of the marginal means suggested that the effect of scene regularity was significant for both CA and CD (for CA: t(9)=10.5 *p*<.0001; Cohen's d=3.74; for CD t(9)=5.78 *p*<.0001; Cohen's d=1.66), but that CA showed a larger effect.

The data are consistent with previous reports that changes associated with appearance of objects in the scene are easier to detect than those associated with disappearances (Cervantes Constantino et al., 2012; Sohoglu and Chait, 2016b; Eramudugolla et al., 2005; Pavani and Turatto, 2008). Given the spectral separation between objects, the steep drop in performance for larger scenes likely linked with the growing computational load of monitoring multiple streams in parallel (rather than inter-component masking). Importantly, the main, novel result is that listeners’ capacity to detect changes (both appearances and disappearances of objects within the scene) depends on temporal regularity.

The **response time** data demonstrated a pattern similar to that for the detection performance. Listeners were slower to detect disappearance (relative to appearance) events, and, importantly, for both CA and CD, reaction times were significantly slower in RAND, relative to REG, scenes. A repeated measures ANOVA revealed main effects of scene regularity (F(1,9)=27.4, *p*=.001; partial η^2^ =0.753 ), change type (F(1,9)=71.55, *p*<.0001; partial η^2^ =0.888) and scene size (F(2,18)=84.99, *p*<.0001; partial η^2^ =0.904) as well as the following interactions: regularity × change type (F(1,9)=20.99, *p*=.001; partial η^2^ =0.7) and change type × scene size (F(2,18)=10.46, *p*=.003; partial η^2^ =0.537). An inspection of the marginal means revealed that the effect of scene regularity was significant for both CA and CD, but that CD showed a larger effect (for CA: t(9)=-2.99 *p*=.015; Cohen's d=0.69; for CD t(9)=-5.6 *p*<.0001; Cohen's d=1.53).

Thus the results replicate the behavioural observations from Sohoglu and Chait (2016a) and further extend them to demonstrate that CD performance is also improved in REG relative to RAND scenes.

## 2 Experiment 1b – the perceptual advantage of regularity extends to spatialized scenes

**Fig. 3:**
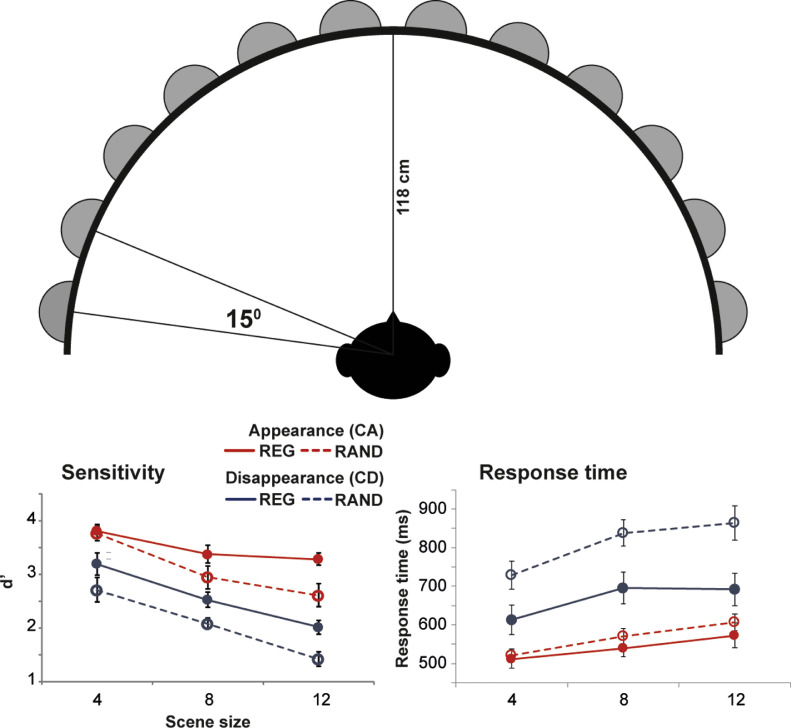
Experiment 1B. [top] Schematic diagram of the loudspeaker array. [bottom] Results of Experiment 1b. Error bars are 1 standard error (SE). In all measures (d’ and response time) performance is significantly reduced in RAND relative to REG scenes.

Here we repeated essentially the same paradigm as in Experiment 1a, but in the context of a spatialized scene. Instead of presenting the signals over headphones where all were co-localized at the center of the head, each stream was presented through a different loudspeaker (12 overall; 15° separation) positioned around the listener (See Fig. 3). This mimicked a more natural listening environment where each scene source is associated with a distinct spatial location. Will the previously observed regularity advantage extend to such settings?

### 2.1 Materials methods & procedure

#### 2.1.1 Stimuli

The stimuli were as in Experiment 1, except for the following differences: The experiment was conducted in an anechoic chamber (IAC, Winchester, UK). Listeners sat in the center of a 12- loudspeaker array (see Fig. 3), with loudspeakers arranged at 15° spacing on the horizontal plain at the level of the listener's ears. Participants were instructed to fixate at a cross drawn between the two front-most loudspeakers. Scene sizes of 4, 8 or 12 concurrent streams were used.

Scenes were generated randomly as before except that, in addition, the location of each stream was randomly assigned to one of the 12 loudspeakers. Therefore, on each trial, the occurrence of a change, the identity of the changing stream, or its location, were unpredictable. Trial numbers were as in Exp1a.

#### 2.1.2 Analysis

Dependent measures are d’ scores and response times. The α level was *a priori* set to 0.05.

#### 2.1.3 Participants

Ten new paid participants took part in the experiment (7 female; mean age = 23.9 years). All reported normal hearing and no history of neurological or audiological disorder.

### 2.2 Results

Fig. 3 shows change detection performance. A repeated measures ANOVA on d’ data with scene regularity, change type and scene size as factors revealed main effects of scene regularity (F(1,9)=22.9, *p*=.001; partial η^2^ =0.718), change type (F(1,9)=218.86, *p*<.0001; partial η^2^ =0.961) and scene size (F(2,18)=35.96, *p*<.0001; partial η^2^ =0.800), with no interactions.

To directly compare with Experiment 1a, we also conducted a second ANOVA with experiment (Exp 1a, vs. Exp 1b) as a between subjects factor. In addition to identical main effects to those detailed above, the analysis revealed a main effect of experiment (F(1,18)=9.95 *p*=.005; partial η^2^ =0.356) and an interaction of scene regularity by change type by experiment (F(1,18)=7.77 *p*=.012 ; partial η^2^ =0.302). This interaction stemmed from the fact that whilst the effect of regularity on CD performance did not differ between experiments (F(1,18)=0.57 *p*=.462 ; partial η^2^ =0.03), that on CA was smaller in the spatialized scenes relative to those in Experiment 1a (F(1,18)=10.52 *p*=.005 ; partial η^2^ =0.369). A likely explanation for this effect is that the spatialization of the scene resulted in better CA change detection overall (significantly better CA performance in RAND scenes in Exp 1b relative to Exp1a; F(1,18)=18.76 *p*<.0001 ; partial η^2^ =0.510) leaving less room for improvement with the introduction of regularity.

Turning to RT: a repeated measures ANOVA on RT data revealed main effects of scene type (F(1,18)=60.68, *p*<.0001; ; partial η^2^ =0.771), change type (F(1,18)=149.58, *p*<.0001; partial η^2^ =0.893) and scene size (F(2,36)=139.46, *p*<.0001; partial η^2^ =0.886) as well as the following interaction: scene size x experiment (F(2,36)=5.15, *p*=.036; partial η^2^ =0.222).

On the whole the results suggest that, though spatialization made certain changes (specifically, CA) overall easier to detect, the advantage of regularity persists also in scenes where streams are widely spatially distributed.

## 3 Experiment 2a,b - the perceptual advantage of regularity extends to complex temporal patterns

**Fig. 4:**
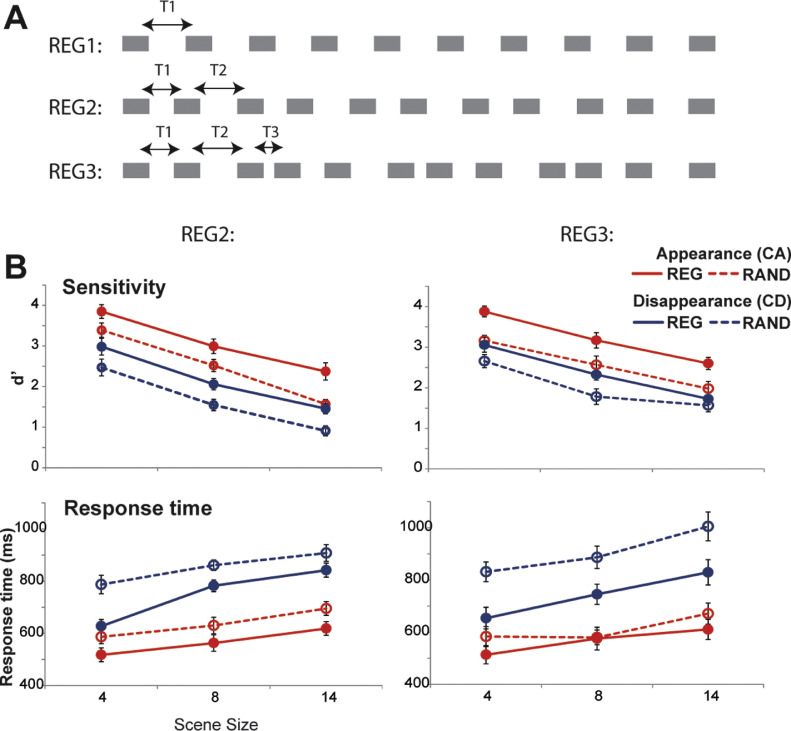
Experiments 2a and 2b. [A] Schematic representations of the regular patterns used. Scene streams in Experiment 1 (REG1) contained a fixed inter-tone-interval (T1) that was randomly chosen for each stream in each trial. Those in Experiment 2a (REG2) contained two different, regularly repeating, inter-tone-intervals (T1 and T2). T1 and T2 were randomly chosen for each stream in each trial. REG patterns in Experiment 2b (REG3) contained three different, regularly repeating, inter-tone-intervals (T1, T2 and T3). These were randomly chosen for each stream in each trial. [B] Results of Experiment 2a (Left) and Experiment 2b (Right) expressed in terms of d’ scores (top) and reaction times (bottom). Error bars are 1 standard error (SE). As in Experiment 1, performance is significantly increased in REG relative to RAND scenes.

Experiment 1 used simple isochronous patterns. Here we ask whether an advantage of regularity will also extend to more intricate patterns. Towards this aim we created non isochronous, repeating patterns (Fig. 4) which model increasingly complex temporal regularities.

### 3.1 Materials and methods

#### 3.1.1 Stimuli

Stimuli were identical to those in Experiment 1, above, except that REG scenes were characterized by increasingly complex temporal patterns. In **Experiment 2**a REG streams were constructed by randomly choosing two inter-tone-interval durations (T1, and T2 in Fig. 4A; values uniformly distributed between 20 and 160 ms; different for each stream) which then repeated regularly. Henceforth, this condition will be referred to as REG2. In **Experiment 2**b scene streams contained three randomly selected, regularly repeating inter-tone-intervals. This condition is referred to as REG3. In both experiments, only inter-tone intervals were manipulated. Tone-pip durations were randomly chosen for each stream, from within the same range as above, and then fixed for the duration of the stimulus. Inter-tone-interval durations and tone-pip durations were chosen anew for each stream in each trial. Overall 45 trials of each stimulus condition were presented.

#### 3.1.2 Participants

Eleven subjects (mean age 22.9 years; 7 females) participated in Experiment 2a and 10 subjects (mean age 22.1; 9 females) participated in Experiment 2b. An additional participant was excluded from the analysis due to very low performance scores (d’=1.5 in the easiest condition [REG CA scene size 4]; for the rest of the subjects d’>3.3). This exclusion did not affect the results in any way. Four subjects participated in both experiments. None participated in Experiment 1.

### 3.2 Results

Results are presented in Fig. 4. Performance in both experiments resembled that in Experiment 1. REG2: a repeated measures ANOVA on **d’** with scene regularity (REG vs. RAND), change type (CA vs. CD) and scene size (4, 8, or 14 streams) as factors revealed main effects of regularity (F(1,10)=32.3 *p*<.0001;partial η^2^ =0.764), change type (F(1,10)=92.6 *p*<.0001) and scene size (F(2,20)=166.5 *p*<.0001; partial η^2^ =0.903) with no interactions. An identical pattern is observed for REG3: main effects of regularity (F(1,9)=16.6 *p*=.003; partial η^2^ =0.649), change type (F(1,9)=79.6 *p*<.0001; partial η^2^ =0.898) and scene size (F(2,18)=82.9 *p*<.0001; partial η^2^ =0.902). We also observed a marginally significant interaction between regularity and change type F(1,9)=5.51 *p*=.044; partial η^2^ =0.380; due to an overall larger effect of regularity on CA relative to CD).

**Response time** data similarly revealed effects comparable to those in Experiment 1. REG2: a repeated measures ANOVA on RT data revealed main effects of regularity (F(1,10)=28.87 *p*<.0001; partial η^2^ =0.743), change type (F(1,10)=88.1 *p*<.0001; partial η^2^ =0.898) and scene size (F(2,20)=39.67 *p*<.0001; partial η^2^ =0.799) with no interactions. REG3: main effects of regularity (F(1,9)=85 *p*<.0001 ; partial η^2^ =0.904), change type (F(1,9)=351.6 *p*<.0001; partial η^2^ =0.975) and scene size (F(2,18)=32.5 *p*<.0001; partial η^2^ =0.783) as well as the following interactions: change type x scene size (F(2,18)=11 *p*=.002; partial η^2^ =0.552; due to CD showing larger decline in performance with scene size than CA) and regularity x change type (F(1,9)=17.3 *p*=.002; partial η^2^ =0.658). An inspection of the marginal means revealed that the effect of scene regularity was significant for both CA and CD, but that CD showed a larger effect (for CA: t(9)=-3.44 *p*=.007; Cohen's d=0.39; for CD t(9)=-7.35 *p*<.0001; Cohen's d=1.43).

Overall, the data demonstrate that, on all measures, change detection performance was significantly improved in REG relative to RAND scenes. This suggests that listeners are able to track complex regular patterns associated with multiple simultaneous acoustic streams.

To understand whether performance varies with the complexity of the regularity, an across group ANOVA was conducted to compare performance across Experiment 1 (‘REG1’), Experiment 2a ('REG2') and Experiment 2b ('REG3'). For d’ this revealed main effects of scene regularity (F(1,28)=106.48 *p*<.0001; partial η^2^ =0.792), change type (F(1,28)=203.14 *p*<.0001; partial η^2^ =0.879) and scene size (F(2,56)=429.28 *p*<.0001; partial η^2^ =0.939) and an interaction between regularity and change type (F(1,28)=9.01 *p*=.006; partial η^2^ =0.243). An inspection of the marginal means suggested that the interaction stems for a larger effect of regularity on CA relative to CD trials. There was no main effect of experimental group, or interaction involving this factor.

A similar test for RT demonstrated main effects of scene regularity (F(1,28)=106.30 *p*<.0001; partial η^2^ =0.792), change type (F(1,28)=347.54 *p*<.0001; partial η^2^ =0.925) and scene size (F(2,56)=128.01 *p*<.0001; partial η^2^ =0.821) as well as the following interactions: change type x scene size (F(2,28)=17.84 *p*<.0001; partial η^2^ =0.389); regularity x change type (F(2,28)=38.96 *p*<.0001; partial η^2^ =0.582); regularity x change type x experimental group (F(2,28)=4.378 *p*=.019; partial η^2^ =0.246).

These results suggest that the complexity of the regularity (i.e., isochronous vs. more complex temporal patterning) did not affect change detection performance. In all cases, a similar improvement was observed for REG relative to the RAND scenes.

## 4 Experiment 3 – changing stream- and context- regularity contribute independently to improved performance

Next, we sought to determine whether the effect of regularity is driven by sensitivity to the temporal regularity of the **changing stream** (appearing or disappearing) per se, or by that of the **context** (the other, non-changing, scene elements). We reasoned earlier that sensitivity to context regularity is key for detecting appearance events (CA). This is because the high predictability of the unfolding REG scene renders the onset of new, unexpected, streams particularly salient. However, since CA detection can, in principle, be based on the first transient associated with the onset of the new stream, it may be that stream regularity as such does not affect performance. In contrast, we expect that for item disappearance (CD), sensitivity to stream regularity should be of key importance because, as discussed above, detection of the cessation of a stream can be vastly improved by tracking its temporal structure.

To understand the effects of changing stream and context we systematically de-coupled the two factors by creating scenes in which the regularity of the changing stream (appearing or disappearing) is independent of the regularity of the rest of the scene.

### 4.1 Martials and methods

#### 4.1.1 Stimuli

‘Regular’ (REG) and ‘Random’ (RAND) scenes were created as before (Experiment 1) with the exception that the regularity of the **changing stream** stream (appearing or disappearing) was manipulated independently of the regularity of the rest of the streams in the scene ('**context**'), resulting in 4 configurations for each change type (CA, CD or NC): REG_REG, REG_RAND, RAND_REG and RAND_RAND (in each case the first term refers to the regularity of the scene context, the second to the regularity status of the changing stream). Two scene sizes – 8 and 14 – were used. Stimuli were blocked by context and change type (CA vs. CD). Overall 45 trials of each stimulus condition were presented.

#### 4.1.2 Participants

Ten new participants (6 females; mean age = 29.4 years) took part in the experiment.

### 4.2 Results

**Fig. 5:**
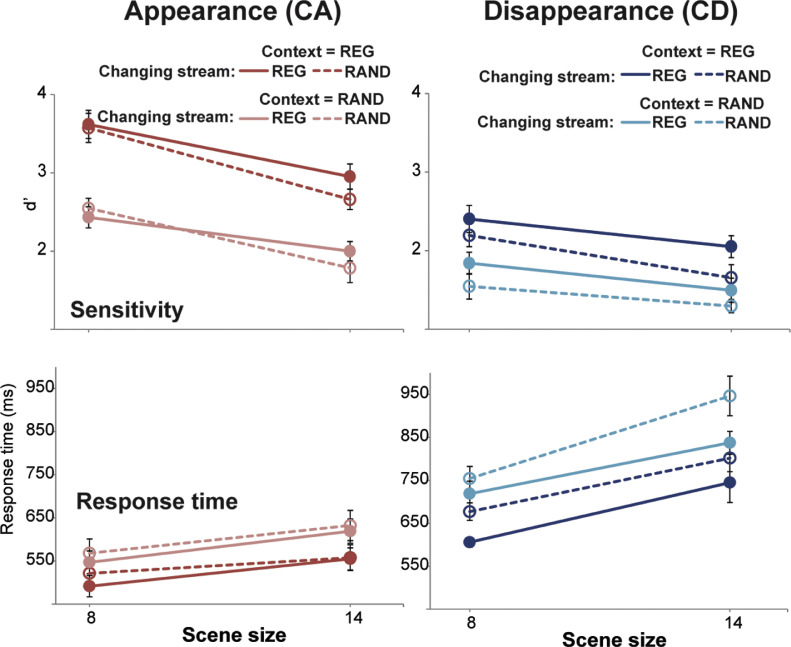
Results of Experiment 3. Appearance changes (CA; red colors) are on the left and disappearance changes (CD; blue colors) are on the right. REG context conditions are in darker colors; RAND context conditions are in lighter colors. REG changing-stream conditions are plotted with solid lines; RAND chaing-stream conditions are plotted with dashed lines. Error bars are 1 standard error (SE). The regularity of the **changing stream** as well as that of the **context**(non-changing) streams both contribute independently to the advantage of regularity (For interpretation of the references to color in this figure legend, the reader is referred to the web version of this article.)

Results are in Fig. 5. A repeated measures ANOVA on **d’** data, with context regularity (REG vs. RAND), changing-stream regularity (REG vs. RAND), change type (CA vs. CD) and scene size (8 or 14 streams) as factors, showed main effects of context regularity (F(1,9)=90.65 *p*<.0001; partial η^2^ =0.910), changing-stream regularity (F(1,9)=20.27 *p*=.001; partial η^2^ =0.692), change type (F(1,9)=64.94 *p*<.0001; partial η^2^ =0.878) and scene size (F(1,9)=82.21 *p*<.0001; partial η^2^ =0.901) as well as the following interactions: change type x scene size (F(1,9)=6.13 *p*=.035; partial η^2^ =0.405 also seen in Experiment 1) and context regularity x change type (F(1,9)=24.52 *p*=.001; partial η^2^ =0.731). An inspection of the marginal means revealed that the effect of context regularity was significant for both CA and CD, but that CA showed a larger effect (for CA: t(9)=10.885 *p*<.0001; Cohen's d=2.99; for CD t(9)=5.574 *p*<.0001; Cohen's d=1.401).

For **response times**, a repeated measures ANOVA showed main effects of context regularity (F(1,9)=68.44 *p*<.0001; partial η^2^ =0.884), changing-stream regularity (F(1,9)=26.20 *p*=.001; partial η^2^ =0.744), change type (F(1,9)=98.43 *p*<.0001; partial η^2^ =0.916) and scene size (F(1,9)=53.63 *p*<.0001; partial η^2^ =0.856) as well as the following interactions: change type x scene size (F(1,9)=16.3 *p*=.003; partial η^2^ =0.644 also seen in Experiment 1, above, and due to CD showing larger decline in performance with scene size than CA) and changing-stream regularity x change type (F(1,9)=10.8 *p*=.009; partial η^2^ =0.546). An inspection of the marginal means revealed that the effect of changing-stream regularity was significant for both CA and CD, but that CD showed a larger effect (for CA: t(9)=-4.23 *p*=.002; Cohen's d=0.69; for CD t(9)=-4.87 *p*=.001; Cohen's d=1.47).

Overall the data suggest that both changing-stream- and context- regularity independently affected change detection performance. But that context regularity had a stronger effect on CA and changing-stream regularity had a stronger effect on CD. This is consistent with the hypothesis that the detectability of CA depends primarily on the first event within the appearing stream (and as such shouldn't be affected by the patterning of the sequence). In contrast, successful coding of temporal structure is critical for the rapid detection of stream disappearance. Indeed, to efficiently determine that a stream has disappeared from the scene, an ideal observer must ‘acquire’ the pattern of onsets and offsets associated with that channel, and respond as soon as an expected tone pip fails to arrive. Importantly, since the identity of the changing stream varied randomly from trial to trial, to achieve optimal performance one must be able to represent the temporal structure of *all objects* within the scene. That listeners were indeed consistently better at detecting CD events in REG scenes demonstrates that, listeners do, at least to some extent, acquire the temporal structure of all (or at least a subset of) on-going scene elements and use this information during scene perception.

## 5 Experiment 4 – REG streams in a RAND context do not pop out

The design of experiment 3 was possibly confounded in the sense that regular streams in a random context might have perceptually stood out, even before the actual change event, thus facilitating the scanning for possible changes and leading to the changing-stream effects observed above.

Here we investigated the extent to which listeners are sensitive to such situations: do REG streams in a RAND context (or vice versa - RAND streams in REG scenes) pop out?

### 5.1 Materials and methods

#### 5.1.1 Stimuli

**Fig. 6:**
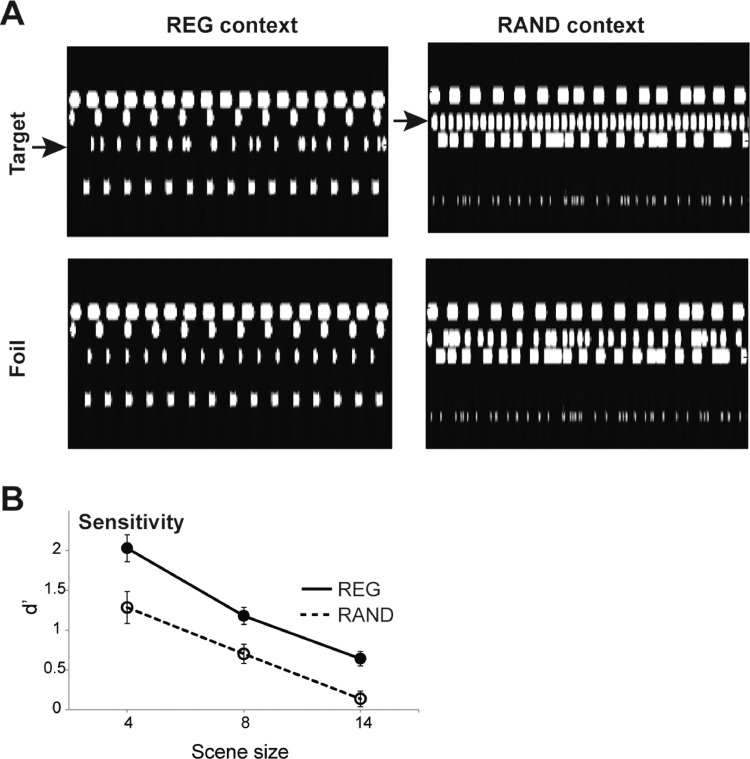
Experiment 4. [A] An Example of REG and RAND context scenes (left and right, respectively) with 4 streams. ‘Foil’ scenes (bottom) contain all REG or all RAND streams; ‘Target’ scenes (top) contain an odd stream – regular among random or vice versa, indicated with arrows. The plots represent ‘auditory’ spectrograms, generated with a filter bank of 1/ERB wide channels equally spaced on a scale of ERB-rate. Channels are smoothed to obtain a temporal resolution similar to the Equivalent Rectangular Duration. [B] Results of Experiment 4. The REG context condition is plotted with a solid line, the RAND context condition is plotted with a dashed line. The results demonstrate that it is consistently easier to detect a random stream among regular streams (REG context) than vice versa.

This experiment used only NC stimuli. ‘REG context’ scenes contained all regular streams (‘Foil scenes’) or one random stream among regular streams (‘target’ scenes’; 50%). Conversely, ‘RAND context’ scenes contained all random streams or (in 50% of the signals) one regular stream among random streams (‘target’ scenes). Within each context condition, scenes were generated in target/foil pairs such that each duo consisted of identical streams (in terms of frequency and temporal properties) only differing by the temporal structure of the target stream (See Fig. 6A). The stimuli were then presented to the listeners in random order, blocked by context type (REG or RAND). Three scene sizes (4, 8 and 14 streams) were used. Participants were instructed to detect the odd streams (regular among random or vice versa – ‘target’ scenes). Overall 45 trials of each stimulus condition were presented.

#### 5.1.2 Participants

Nine new subjects participated in the experiment (4 female; mean age = 28.7 years). The data from an additional participant were not useable due to a technical error.

### 5.2 Results

Fig. 6B shows the results of Experiment 4. A repeated measures ANOVA on **d’** scores, with context (REG versus RAND) and scene size (4, 8 or 14 objects) as factors, revealed main effects of context (F(1,8)=37.8, *p*<.001; partial η^2^ =0.825) and scene size (F(2,16)=52.3, *p*<.001; partial η^2^ =0.867) with no interactions.

The relatively steep decline with scene size suggests that regularity does not strictly ‘pop-out’ but is rather discovered via some search-based process. That it is overall easier to find a random stream in a REG context, rather than a regular stream in a RAND context, is in line with the theoretical account proposed above: listeners acquire the temporal patterning associated with each stream in a REG context scene such that events that do not conform with these patterns (i.e those belonging to the belonging RAND stream) are relatively easy to detect. Conversely, in a RAND context, where most events are unpredictable, it is more difficult to spot the one stream that follows a regular pattern.

Importantly, the results demonstrate that at the largest scene size (14) participants are at floor for detecting a regular stream in a RAND context (one sample t test against 0: t=1.4, *p*=.19). Combined with the findings from Experiment 3, above, the data demonstrate that despite not being aware of the regularity of the changing stream, participants implicitly used this information for change detection: even with 14 concurrent streams in the scene, listeners’ change detection performance benefitted when a disappearing stream was regular, relative to when it was random. Indeed, all the results from Experiment 3, both in terms of d’ and RT, remain significant when running the ANOVA on scene size 14 only (*p*<.01 for all). This reveals that even in very crowded scenes, participants are able to utilize temporal regularity, of which they are not consciously aware, to efficiently detect change events.

## 6 General discussion

The auditory system is tuned to changes in the acoustic environment (Cervantes Constantino et al., 2012; Sohoglu and Chait, 2016b; Eramudugolla et al., 2005; Pavani and Turatto, 2008). We therefore chose a change detection task as a neuro-ethologically relevant means by which the role of sensitivity to temporal structure in the course of auditory scene analysis can be studied. Mounting evidence suggests that listeners are sensitive to the temporal structure of sound sequences and use this information to anticipate and improve their interaction with expected events, even in the absence of directed attention (Andreou et al., 2015; Jones et al., 2002; Geiser et al., 2012). Here we show that the auditory system's ability to track the temporal structure of on-going sound input and register when it is violated persists even when the scene is heavily populated with concurrent streams and the identity of the changing component is in advance unknown.

Specifically, we demonstrate that listeners’ change detection ability is facilitated when scene streams are characterized by a temporally regular fluctuation pattern (Experiment 1). The regularity of the changing stream (appearing or disappearing) as well as that of the background (non-changing) streams both contribute independently to this effect (Experiment 3). The advantage of regularity, relative to random temporal patterning, is observed even when using complex, non-isochronous, temporal patterns (Experiment 2). These findings establish that perception of complex acoustic scenes relies on the availability of detailed representations of the regularities automatically extracted from each scene stream.

### 6.1 Are listeners coding local (frequency specific) or global temporal regularities?

An important point concerns whether the patterns were detected within each frequency channel separately, or identified as a complex, temporal regularity across the entire frequency range. In the present stimuli, local regularity is intrinsically linked to global regularity and it is therefore difficult to dissociate the two. However, key elements of the paradigm, including the use of multiple, random-phase streams that are widely spaced in frequency, were explicitly implemented to encourage listeners to process the signals as multiple concurrent stream (Shamma et al., 2011; see also Brochard et al., 1999; Demany et al., 2015; Sussman et al., 2005). Because the regular patterns characterizing each stream are simple, compared with the much more complex aggregate pattern, it may be reasonable to conclude that patterns were extracted within each component separately. Several key observations support this assertion: (a) The advantage of regularity persisted even for complex temporal patterns (Experiment 2). (b) The advantage of regularity persisted in the face of spatial separation between streams (Experiment 1b), which presumably helped to increase stream distinctiveness. (c) The ‘changing-stream’ effect in Experiment 3 - listeners exhibited improved performance when the changing stream was regular even when the rest of the streams in the scene were random. Conversely, they showed reduced performance when the changing stream was random in an otherwise regular scene.

### 6.2 Do regular patterns attract attention?

It has been suggested that attention can be understood as a process that infers the level of predictability of sensory signals such that highly predictable sensory streams capture attention in a bottom-up manner (Jones et al., 2002; Feldman and Friston 2010; Auksztulewicz and Friston, 2015). In contrast, here (Experiment 4) we show that regular patterns in a background of random patterns do not pop-out and are in fact always harder to detect than vice versa. On the whole, the results suggest that while sensitivity to regularity plays a key role in shaping our perception of our surroundings, this does not translate to explicit attentional capture (Southwell et al., 2017; Meijs et al., 2018).

Consistent with this, we demonstrate that listeners benefitted from regularity despite not being consciously aware of it: Listeners were at floor when asked to determine whether a regular stream was present in a scene containing 14 concurrent random streams (Experiment 4), but exhibited a sizeable improvement to change detection performance when that stream disappeared (Experiment 3). This suggests that the temporal structure of that stream was automatically tracked by the auditory system and used to facilitate scene analysis. This finding is in line with a previous demonstration in the visual modality (Zhao et al., 2013): A visual search task was facilitated at a location which previously contained a regularity. This occurred even though participants reported not being aware of the regular pattern.

### 6.3 Sensitivity to temporal regularity in the service of auditory scene analysis

In an MEG study, Sohoglu and Chait (2016a) recorded responses to REG and RAND scenes in the context of an appearance (CA) detection task. The behavioural advantage associated with REG scenes was accompanied by increased responses in auditory cortex and parietal cortex both before, as well as after, the change. This was interpreted as reflecting the operation of mechanisms which rapidly infer the precision (predictability) of sensory input and upregulate responses to reliable sensory information, such that violations of these patterns (e.g., in the form of an appearing or disappearing streams) evoke higher prediction errors (see also Southwell and Chait, 2018).

The behavioural effects observed here support this interpretation: The ‘context’ effects shown in Experiment 3 (where listeners were better at detecting changes in scenes where the ‘background, non-changing, streams were regular) and the demonstration that listeners are consistently better at spotting random sequences within scenes that otherwise comprised of regular components, than vice versa (Experiment 4) demonstrate increased sensitivity to deviants in REG than RAND context.

In all, these results demonstrate that the auditory system rapidly discovers regular structure in the unfolding sensory input, in line with a broader theoretical framework which views the brain as a regularity extractor (Winkler et al., 2009; Arnal and Girad, 2012; Hohwy, 2013). Whilst regular structure per se, does not attract attention it is monitored automatically and used to facilitate listening. Transients (onsets of individual tones) in regular streams are predictable and thus do not require substantial processing resources, making it easier to ignore regular patterns when these are task irrelevant (Andreou et al., 2011; Southwell et al., 2017). On the other hand, unexpected events, including the non-arrival of an expected sound, in an otherwise predictable stream are rendered as ‘surprising’ or ‘salient’ and capture bottom-up attention (Southwell and Chait, 2018; Kaya and Elhilali, 2014).

### 6.4 Neural underpinnings

Source analysis in Sohoglu and Chait (2016a) suggested that scene temporal structure modulated neural responses in a network of brain regions, including auditory regions in the superior temporal lobe and the left post central gyrus consistent with accumulating evidence which has implicated the left parietal cortex in temporal processing (Andreou et al., 2015; Coull and Nobre, 2008; Coull, 2009).

Various mechanisms have been proposed to account for temporal structure learning (Coull, 2009; Grahn, 2012; Merchant et al., 2013) though these are usually based on paradigms with slower dynamics than those here. Currently in receipt of substantial attention are a family of oscillatory models (e.g., Arnal and Girad, 2012) according to which the brain entrains to the temporal structure of the auditory input and the resulting periodic increases in excitability underlie the behavioural effects of temporal regularity. A challenge may be to extend such mechanisms to non-isochronous regular sequences as used here. One possibility is that sensitivity to complex temporal regularities is supported by an array of oscillators, which share a period but differ in phase. Alternatively, the observed learning could be accomplished within interval timing mechanisms (Merchant et al., 2013) which explicitly code interval duration.

It is remarkable that listeners in the present experiments exhibited the ability to track the regularity of individual streams even in very crowded scenes, populated by up to 14 simultaneous sound streams. That normal, young, listeners rely on this capacity so routinely, makes it an interesting feature to investigate in certain clinical populations typically linked to failure to extract temporal regularities (Schapiro et al., 2014; Ciullo et al., 2018) as well as during healthy aging (Rimmele et al., 2012; de Kerangal et al., 2020). The simplicity of the present paradigm makes it easily extendible to animal models allowing a cross species and systems-level investigation of the observed effects.

## CRediT authorship contribution statement

**Lucie Aman:** Formal analysis, Investigation, Writing - original draft. **Samantha Picken:** Formal analysis, Investigation, Writing - original draft. **Lefkothea-Vasiliki Andreou:** Formal analysis, Investigation, Writing - original draft. **Maria Chait:** Conceptualization, Data curation, Funding acquisition, Methodology, Project administration, Supervision, Writing - original draft.

## Appendix Supplementary materials

Image, application 1
